# Relative-to-resident abuse in Norwegian nursing homes: a cross-sectional exploratory study

**DOI:** 10.1186/s12877-024-05513-0

**Published:** 2024-11-05

**Authors:** Anja Botngård, Arne Henning Eide, Laura Mosqueda, Lene Blekken, Wenche Malmedal

**Affiliations:** 1 Department of Public Health and Nursing, Norwegian University of Science and Technology, Post box 8905, Trondheim, N-7491 Norway; 2 Department of Health Research, SINTEF Digital, Oslo, Norway; 3 Department of Social Work, Norwegian University of Science and Technology, Trondheim, Norway; 4 Department of Family Medicine, Keck School of Medicine, University of Southern California, Los Angeles, USA

**Keywords:** Relative-to-resident abuse, Nursing homes, Institutional settings, Non-staff abuse, Elder abuse

## Abstract

**Background:**

In community settings, relatives often provide care to their older family members, which is sometimes perceived as a high burden, overwhelming and stressful, contributing to an increased risk of elder abuse. In most countries, relatives have no legal obligation to provide care when family members are admitted to nursing homes; nevertheless, studies have shown that relatives continue to provide emotional, instrumental, and personal care after admission, often related to the understaffing and high workload of nursing staff. Despite the growing interest in elder abuse in nursing homes, most studies have concentrated on the abuse perpetrated by nursing staff or co-residents, but few studies have explored the abuse that relatives may perpetrate.

**Methods:**

This study was a cross-sectional survey of 3,693 nursing staff members recruited from 100 nursing homes in Norway, to examine the extent of relative-to-resident abuse in Norwegian nursing homes, as observed by nursing staff.

**Results:**

The findings indicate that 45.6% of the nursing staff had observed one or more episodes of relative-to-resident abuse during the past year. Among the subtypes of abuse, 44.8% of the nursing staff had observed psychological abuse, 8.4% had observed physical abuse, 2.7% had observed financial/material abuse, and 0.7% had observed sexual abuse at least once during the past year.

**Conclusions:**

This is the first large study exploring the extent of relative-to-resident abuse in nursing homes, which is a phenomenon that is significantly less addressed than abuse committed by staff and co-residents. The findings in our study illustrate that abuse committed by relatives needs more awareness and attention to improve the well-being of nursing home residents. Further research is recommended to enhance our understanding of such abuse and should include other approaches measuring the proportion of relative-to-resident abuse, as relying solely on staff observations is insufficient for determining the prevalence in this case. Future studies should also examine the cumulative impact of victimization in nursing homes and should include an analysis of how cases of abuse are reported and handled.

## Background

Elder abuse can be defined as “an intentional act or failure to act by a caregiver or another person in a relationship involving an expectation of trust that causes or creates a risk of harm to an older adult” and includes physical, psychological, financial/material, sexual abuse, and neglect [1]. The impact of elder abuse on victims can be significant, with consequences ranging from various psychological and physical symptoms to premature mortality [2]. The societal implications of elder abuse can also be substantial, with increased utilization of health care services, including hospitalizations, and greater usage of behavioural health services, imposing financial strain on society [2]. Older adults may be exposed to abuse in community or institutional settings, where perpetrators can be spouses/partners, children, grandchildren, healthcare providers, others in close relationships, or co-residents in nursing homes. Several studies have investigated the scope of elder abuse perpetrated by relatives in community settings and by staff and co-residents in nursing homes. Few studies have examined the extent of abuse perpetrated by relatives in nursing homes, which was the aim of the current cross-sectional study of nursing staff in Norwegian nursing homes.

In community settings, meta-analyses have estimated that the prevalence of elder abuse ranges from 15.7 to 34.3%, with psychological abuse being the most reported [3–5]. In Norway, a study of older adults in community settings estimated the prevalence of elder abuse to be between 6.8% and 7.2%, where most perpetrators were in close contact with the victims [6]. This study did, however, not include individuals with cognitive impairments, those over the age of 90, or non-Norwegian speakers. A more recent study in Norway that examined the occurrence of abuse committed by informal caregivers towards home-dwelling persons with dementia revealed that 66.3% of caregivers had been involved in some form of abusive episode during the past year [7]. The most common type of abuse was psychological abuse, followed by physical, financial, and sexual abuse [7].

In institutional settings, studies show that approximately 60% of staff members commit one or more episodes of staff-to-resident abuse each year, with the highest rates reported for psychological abuse [8, 9]. In the same settings, studies have also indicated that residents may be exposed to aggression by their co-residents [10–12]. In the United States, Zhang et al. [13] conducted a survey of family members on the extent of abuse and exploitation by staff and non-staff (co-residents, visitors, family members) in Michigan nursing homes. They reported that 36.5% of family members reported cases of staff abuse, while 10% reported cases of non-staff abuse [13].

Elder abuse, a complex issue with many facets, is often examined from a socioecological perspective that emphasizes various risk factors across several levels: individual (victim and perpetrator), relational, community, and societal [14]. In community settings, studies on elder abuse have identified several individual risk factors for both victims and perpetrators of abuse, including physical and mental health issues, substance abuse, negative attitudes, and prior victimization [15, 16]. At the relational level, research has indicated that a perpetrator’s dependency on victims or vice versa can be a risk factor, in addition to other relational issues such as the inability to form and maintain positive, romantic, and nonintimate relationships and the presence of conflictual relationships [15]. Studies have also shown that a less satisfactory caring relationship in the past increases the risk of abuse in later caregiving relationships [16]. Relatives often provide care for their older family members living at home, and sometimes this responsibility is perceived as overwhelming and stressful, which may increase the likelihood of elder abuse [16]. At the community and societal levels, factors such as inadequate social support, cultural norms, and ageism have been recognized as contributors to elder abuse [15–17].

In most countries, relatives are not legally required to care for their family members when admitted to a nursing home. However, an interpretative synthesis of the literature on family involvement in nursing homes revealed that relatives continue to provide emotional, instrumental, and personal care after admission [18]. Family members’ participation in this care is generally highly regarded because they possess knowledge of their relatives’ personal history and preferences, enabling effective collaboration with staff in providing care for residents [18]. Despite these advantages, Hovenga et al. [18] found that family members’ involvement in care may also be challenging and that various relative-staff relations, psychosocial and organizational factors may influence this involvement negatively. The relative-staff factors included challenges faced by relatives in redefining their caregiving role, dissatisfaction with the care provided in the nursing home, and perceived incompetence of the nursing staff [18]. Psychosocial factors include feelings of guilt or loneliness experienced by relatives when family members are admitted to nursing homes or difficulties adjusting to changes in their relationships [18]. Organizational factors such as understaffing, high staff turnover and unfriendly staff experiences also contribute to influence family members’ involvement in nursing homes [18].

Buzgova & Ivanova [19] conducted a qualitative study of staff and residents in institutional settings to gain insights into elder abuse committed by different perpetrators, such as staff, relatives, and others. In interviews with staff, they reported cases where relatives had disregarded their family members’ opinions and decisions, especially related to admission to the facility. The staff also mentioned situations where relatives took money on pension day or refused to buy necessary items for the resident. Signs of physical abuse were also observed by staff upon admission to the facility. In interviews with residents, they expressed embarrassment about their children’s abusive behaviour but still appreciated their visits to the facility [19].

Elder abuse is recognized as a public health and human rights problem that is expected to increase with the increasing population of older adults [17]. In a policy brief by the United Nations outlining five priorities for the UN Decade of Health Ageing (2021–2030), one of the key priorities is to enhance the understanding of the prevalence and risk factors associated with elder abuse [17]. While several studies have investigated elder abuse and aggression committed by staff and co-residents, few studies have explored the scope of relative-to-resident abuse in institutional settings. The present study aimed to examine the extent of relative-to-resident abuse in Norwegian nursing homes, as observed by nursing staff.

## Methods

### Study design

This study was part of a larger cross-sectional exploratory study conducted among nursing staff in Norwegian nursing homes between October 2018 and January 2019 and was designed to explore the scope of various types of abuse and aggression, including staff-to-resident abuse [8, 20], resident-to-resident aggression [10], and relative-to-resident abuse.

### Setting

All Norwegian nursing homes (private and public) were eligible for inclusion. In Norway, approximately 90% of nursing homes are owned and managed by municipalities, and 10% are operated by private for-profit or nonprofit organizations [21]. Norwegian nursing homes provide 24-hour care, treatment, and services for patients who require more care than can be provided in home settings and contain both short- and long-term units mainly managed by registered nurses and a physician [22].

### Participants and recruitment

All Norwegian nursing homes are listed in the Central Register of Establishments and Enterprises, and in 2018, there were 939 registered nursing homes. For our study, we randomly selected 100 of these nursing homes (approximately 10%) to participate. Additionally, we randomly selected 50 nursing homes to serve as backups for the initially chosen nursing homes. The inclusion criteria were nursing staff in the participating nursing homes, registered nurses, learning disability nurses/social educators, licenced and practical nurses, health care assistants, and assistants with no formal health education. The exclusion criteria were physiotherapists, occupational therapists, medical doctors, and staff who worked in other units, such as assisted living facilities and daycare centres. In the randomly selected nursing homes, 6,337 staff members were eligible to participate in the study. Of these, 3,811 returned the questionnaires (response rate of 60.1%). We excluded 188 participants because they either did not work directly with residents, worked in assisted living facilities, or did not answer any questions related to abuse, resulting in 3,693 participants and an analytic response rate of 58.3%. Among the 100 nursing homes, response rates varied from a low of 14% to a high of 100%, with a median response rate of 69.3%. Although no direct incentives were provided to the individual participants, we offered a financial incentive to the eight institutions that achieved the highest response rates. Approximately 900 GBPs were allocated for staff welfare in these institutions.

### Data collection

An employee chosen by the nursing home coordinated the survey on-site. This coordinator was provided with a comprehensive instruction letter, survey questionnaires, sealed boxes for the collection of questionnaires, and a prepaid box for returning the sealed collection boxes. Participants were not required to include their names or birth dates on the questionnaire. Consent was considered when nursing staff deposited their completed questionnaires into sealed boxes. After collection, it was not possible to withdraw participation.

### Study variables and measurements

The survey questionnaire consisted of 23 items measuring the frequency of observed abusive acts committed by relatives towards their kin during the past year. These acts were categorized into psychological abuse (7 items), physical abuse (7 items), financial/material abuse (4 items), and sexual abuse (5 items). The response scale ranged from “Never” to “More than 10 times”. The Cronbach’s alpha coefficients were 0.84 for psychological abuse and 0.77 for physical abuse. For financial/material and sexual abuse, the coefficients were below 0.5, possibly due to the skewed results towards “Never”. The survey questionnaire was originally developed in the United States and has been used in several large studies to measure the extent of staff-to-resident abuse and resident-to-resident aggression in institutional settings and assisted living facilities [11, 23–25]. Further details about the original questionnaire, its translation and modification, and the pilot study can be found in a separate article [8].

### Ethical considerations

Participation in the survey was voluntary for both the nursing homes and nursing staff. Nursing home directors who agreed to participate provided written consent to the first author. Informed consent from the nursing staff was obtained upon completion of the survey questionnaire, and the staff members were notified that they could not withdraw their participation after submitting the questionnaire. Each nursing home was assigned a unique code for data analysis, which was securely maintained by the first author. Participants were assured that their identities would remain anonymous in all publications. The study received approval from the Regional Committee for Medical and Health Research Ethics in Norway in May 2018, with Clinical Trial Number: 2018/314.

### Statistical analysis

The data were analysed with Stata software package 17.0. Descriptive statistics, including percentages, means, and standard deviations (SDs), are used to present the participating nursing homes and nursing staff. Observations of abuse from relatives towards residents are presented as percentages. The observations were also dichotomized into “No abuse” (never) and “Abuse” (one or more observations) across all subtypes and are presented as percentages.

## Results

### Characteristics of nursing homes and nursing staff

The participating nursing homes varied in size from eight to 161 beds (mean 46.7, SD 30.6), where 94% were publicly owned and 6% were privately owned. Of the participating nursing staff, 91.5% were female, the mean age was 41.3 years (SD 14.0), 42.9% were licenced practical nurses, 53.9% worked part-time, and 63.7% worked in long-term units (Table 1).

**Table 1:** Characteristics of the participating nursing staff (*N* = 3,693)

Characteristics	*n* (%)	Mean (SD)
**Gender**		
Female	3362 (91.5)	
Male	312 (8.5)	
**Age (years)**		41.3 (14.0)
**Professional occupation**		
Assistant (no formal health education)	997 (27.5)	
Licenced practical nurse	1553 (42.9)	
Registered nurse/social educator/learning disability nurse	1070 (29.6)	
**Working time**		
Full-time (≥ 35 h per week)	1503 (46.1)	
Part-time (< 35 h per week)	1757 (53.9)	
**Working units**		
Long-term care units	2243 (63.7)	
Dementia special care units	766 (21.8)	
Short-term care units	511 (14.5)	

### The extent of observed relative-to-resident abuse in Norwegian nursing homes

Overall, 45.6% (1530/3359) of the nursing staff had observed at least one episode of relative-to-resident abuse during the past year. Among the subtypes of abuse, 44.8% (1557/3473) of staff had observed psychological abuse, 8.4% (299/3542) had observed physical abuse, 2.7% (95/3591) had observed financial/material abuse, and 0.7% (25/3585) had observed sexual abuse at least once in the past year (Table 2).

**Table 2:** Observations of relative-to-resident abuse among nursing staff during the past year (*N* = 3,693)

Type of abuse:		How often observed in the past year (%):					
		**N**	Never	Once	2–5 times	6–10 times	> 10 times
**Psychological**	Yelling	3616	71.9	11.2	12.8	2.7	1.6
	Nasty remarks	3603	82.7	8.3	6.7	1.4	0.9
	Swearing	3623	92.7	3.8	2.5	0.6	0.4
	Humiliating remarks	3586	83.5	8.5	5.7	1.6	0.7
	Arguing	3608	72.1	13.0	11.3	2.3	1.4
	Threatening remarks	3614	95.5	2.6	1.2	0.4	0.3
	Critical remarks	3607	81.2	10.2	6.4	1.4	0.9
**At least one episode of psychological abuse**:		**1557/3473 (44.8%)**					
**Physical**	Pushing, grabbing, or pinching	3599	96.8	1.9	1.0	0.06	0.2
	Pulling hair or kicking	3608	99.4	0.4	0.2	-	0.06
	Purposely hurting	3613	99.7	0.2	0.06	-	0.06
	Throwing things at a resident	3608	99.7	0.2	0.06	-	0.08
	Hitting	3610	99.4	0.4	0.2	0.03	0.06
	Bullying	3606	96.3	2.2	1.0	0.3	0.1
	Behaving aggressively towards a resident	3598	95.6	2.7	1.2	0.2	0.2
**At least one episode of physical abuse**:		**299/3542 (8.4%)**					
**Financial/** **material**	Stealing money	3612	99.7	0.2	0.06	-	-
	Stealing things	3618	99.7	0.1	0.2	-	0.03
	Signing documents without permission	3614	97.9	1.6	0.5	-	0.03
	Destroying a resident’s things	3613	99.8	0.1	0.08	-	0.03
**At least one episode of financial/material abuse**:		**95/3591 (2.7%)**					
**Sexual**	Unwelcome touching	3616	99.6	0.2	0.1	0.03	0.06
	Unwelcome discussion of sexual activity	3611	99.7	0.3	0.03	-	-
	Exposure of a resident’s private body parts	3607	99.9	0.06	0.03	-	-
	Digital penetration (e.g. finger)	3618	99.94	0.06	-	-	-
	Rape	3610	100	-	-	-	-
**At least one episode of sexual abuse**:		**25/3585 (0.7%)**					

## Discussion

This study highlights the significance of relative-to-resident abuse in nursing homes, where almost half (45.6%) of the nursing staff reported observing at least one episode during the past year, with psychological abuse being the most reported. To our knowledge, this is the first large survey exploring the extent of relative-to-resident abuse in nursing homes. Consequently, there are no comparable studies or estimates to our findings.

Despite limited research on the extent and risk factors for relative-to-resident abuse in nursing homes, it is reasonable to suggest that identified risk factors for victims and perpetrators in community settings, such as physical and mental health issues, substance abuse, negative attitudes, and the presence of conflictual relationships [15, 16], remain significant after a family member is admitted to a nursing home. In our study, we did not examine who the perpetrator (relative) of the abusive acts was, but prior research in community settings has found that most perpetrators of elder abuse are close to the victim, such as partners/spouses [6, 26], especially those with caregiving responsibilities [16]. For some people, these caregiving responsibilities may lead to a sustained and escalating situation of stress, which also heightens the risk of abuse due to an inability to cope with or manage stress and burden [27, 28]. Although relatives are not legally required to provide care after admission to the nursing home, some feel obligated to continue to provide care, leading to a continuance of the feeling of stress and burden [18]. Overall, these factors may explain why an already abusive relationship between residents and relatives may continue after admission to the nursing home.

Experiencing multiple, concurrent, or sequential forms of abuse by one or more individuals is referred to as polyvictimization in later life and can have severe negative impacts on both victims and their families [29]. Older adults may also experience multiple instances of victimization throughout their lives, which is associated with poor health [30, 31].

Since many risk factors for being exposed to abuse by different perpetrators are similar, one may assume that some residents could experience multiple forms of abuse from one or more perpetrators in a nursing home context. In Norwegian nursing homes, we found that residents are exposed to abuse and aggression from both staff [8] and co-residents [10], but we did not investigate whether the same residents were exposed to these various forms of abuse and aggression or whether they had experienced abuse before admission to the nursing home. However, a systematic review of resident-to-resident aggression in nursing homes revealed that residents targeted by such aggression were also more likely to be exposed to abuse by staff [32].

In recent decades, numerous studies have explored the prevalence of and risk factors for staff-to-resident abuse in nursing homes. Thus, in a recent scoping review by Hirt et al. [33], a wide range of prevalence estimates and several inconsistencies in associated factors were identified at both the individual and organizational levels. The authors propose a more comprehensive and less specific conceptualization of elder abuse, which encompasses not only staff-to-resident abuse but also resident-to-staff and resident-to-resident abuse. They suggest this broader approach as a foundation for developing interventions aimed at preventing overall elder abuse in nursing homes [33]. Our study shows that this approach should also include relative-to-resident abuse.

Whether perpetrated by healthcare staff, co-residents, relatives, or others, elder abuse in nursing homes requires increased awareness and understanding to effectively address and for prevention efforts.

This cross-sectional study has certain strengths and limitations. The extent of relative-to-resident abuse was measured through observations of the nursing staff. This could have resulted in an overestimation, as multiple staff members working in the same units might have observed and reported the same abusive acts. The study also required the staff to remember episodes from the past year, which could have introduced recall bias. We did not apply a substantive threshold criterion, such as ten or more incidents in the past year, to define psychological abuse, as some other studies have done [34]. It could be argued that our approach might lead to an overestimation of abuse, since minor episodes like “arguing” between relatives and residents may not constitute abuse. However, considering the significant frailty and vulnerability of nursing home residents and the potential power imbalance of relatives and residents, we deemed a single act of abuse sufficient to qualify as abuse.

Several factors may have led to an underestimation of the extent of abuse by relatives towards residents. The likelihood of staff witnessing financial abuse may be low, as relatives with access to residents’ financial assets may be likely to exploit them at home or another location away from the facility. Similarly, the chances of staff observing physical or sexual abuse may also be low, as these incidents are typically committed out of sight to avoid detection and are not usually carried out in the presence of witnesses. Additionally, some incidents may occur when relatives take residents out of the facility for day visits. Furthermore, some staff members might feel a social obligation to refrain from reporting sensitive interactions between spouses or other relatives.

Another limitation is that the survey instrument was designed for measuring staff-to-resident abuse and has not been validated for measuring abuse by relatives. Last, the cross-sectional design does not provide any causal explanation for such abuse. The strengths of this study include the large sample size of nursing homes (*n* = 100) and staff (*n* = 3693), a response rate of approximately 60%, and to our knowledge, this is the first large study measuring the extent of relative-to-resident abuse in nursing homes.

## Conclusions

Our study contributes to the novel understanding of a phenomenon that is significantly less addressed in nursing homes compared to abuse committed by staff and co-residents. Increased attention to relative-to-resident abuse is needed to improve the well-being of nursing home residents. Further research is recommended to enhance our understanding of the abuse of residents by relatives and include other approaches measuring the proportion of relative-to-resident abuse, as relying solely on staff observations is insufficient for determining the prevalence in this case. Future studies should also examine the cumulative impact of victimization in nursing homes, where adults are particularly vulnerable and frail, and should include an analysis of how cases of abuse are reported and handled.

## Data Availability

The survey questionnaire used is available from the corresponding author upon reasonable request. The questionnaire only exists in the Norwegian language. The dataset used and analysed in this study might be provided by the corresponding author if approved by the Regional Committee for Medical Research Ethics Central Norway. Due to the nature of this topic, study participants were not asked to agree for their data to be shared publicly.

## Abbreviations

SD: standard deviation
